# Supplementary material for: A self-stabilized and water-responsive deliverable coenzyme-based polymer binary elastomer adhesive patch for treating oral ulcer
Source: Nat Commun. 2023 Nov 24;14:7707. doi: 10.1038/s41467-023-43571-x (PMC10673908; doi:10.1038/s41467-023-43571-x)
Supplement: Supplementary file 1 — Supplementary information [file 41467_2023_43571_MOESM1_ESM.docx]

Supplementary Information

**A self-stabilized and water-responsive deliverable coenzyme-based polymer binary elastomer adhesive patch for treating oral ulcer**

**Author:**

Chunyan Cui^1,4^, Li Mei^2,4^, Danyang Wang^2^, Pengfei Jia^2^, Qihui Zhou^3^*, Wenguang Liu^1^*

**Affiliation:**

^1^School of Materials Science and Engineering, Tianjin Key Laboratory of Composite and Functional Materials, Tianjin University, Tianjin 300350, China.

^2^ Department of Stomatology, Qingdao University, Qingdao 266021, China.

^3^ School of Rehabilitation Sciences and Engineering, University of Health and Rehabilitation Sciences, Qingdao 266071, China.

^4^These authors contributed equally: Chunyan Cui, Li Mei

*e-mail: [qihuizhou@qdu.edu.cn](mailto:qihuizhou@qdu.edu.cn); [wgliu@tju.edu.cn](mailto:wgliu@tju.edu.cn)

This PDF file includes:

Supplementary Methods

Supplementary Figures 1–33

Supplementary Tables 1–2

Supplementary References 1–12

**Supplementary Methods**

**Materials.** α-Lipoic acid (LA, 99 %, Macklin, Shanghai, China), sodium hydroxide (NaOH, Heowns, Tianjin, China) and ethanol (AR, Jiangtian Chemical, Tianjin, China) were used as received. Chitosan patch was was purchased from Chuangbang Medical and Health Technology (Yunnan, China) Co., Ltd. All the other reagents are of analytical grade and used without further purification. Water used in this work was purified by a Utop ultrapure water machine with a power of 100 W.

**Characterization of PolyLA, PolyLA-Na film and PolyLA-Na/PolyLA-based adhesive patches.** The nuclear magnetic resonance spectra of the LA and PolyLA ethanolic solution were measured in dimethyl sulfoxide-d6 on a Varian Inova-400 MHz NMR Spectrometer, and the nuclear magnetic resonance spectra of the LA-Na and PolyLA-Na aqueous solution were measured in D_2_O on the same equipment. The nuclear magnetic resonance spectra of dry PolyLA, dry PolyLA-Na film and PolyLA-Na/PolyLA-based adhesive patch were measured by solid state NMR on AVANCE Ⅲ HO. UV-Vis absorption spectra of the PolyLA ethanolic solution, PolyLA-Na aqueous solutions, monomer release in artificial saliva and DPPH solutions were recorded using a UV-Vis spectrometer (Genesys 180, Thermo Scientifc). The surface chemical compositions of the PolyLA-Na/PolyLA-based adhesive patches were measured using an X-ray photoelectron spectrometer (Thermo Scientific ESCALAB 250 Xi). A monochromatic Al Kα X-ray was used as an excitation source (hν = 1486.6 eV) running at 15 kV and 150 W. The neutral C1s peak (C-C (H), set at 284.4 eV) was used as a reference for charge correction. The contact angles were measured at room temperature on an optical contact goniometer (Harke-SPCA, China) after 5 µL drop of deionized water was placed carefully on different patch surface for 20 s. The contact angles at different positions of each sample surface were averaged out. The crystal structure of the LA powder, dry PolyLA, dry PolyLA-Na film and PolyLA-Na/PolyLA-based adhesive patches was determined using an XRD (D8 Advanced, Bruker, Germany). XRD data were obtained from 5° to 50° (2θ) using Cu Kα radiation with a scan rate of 1° min^-1^. Raman spectra were recorded on a DXR Microscope with 532 nm excitation.

**Supplementary Figures**


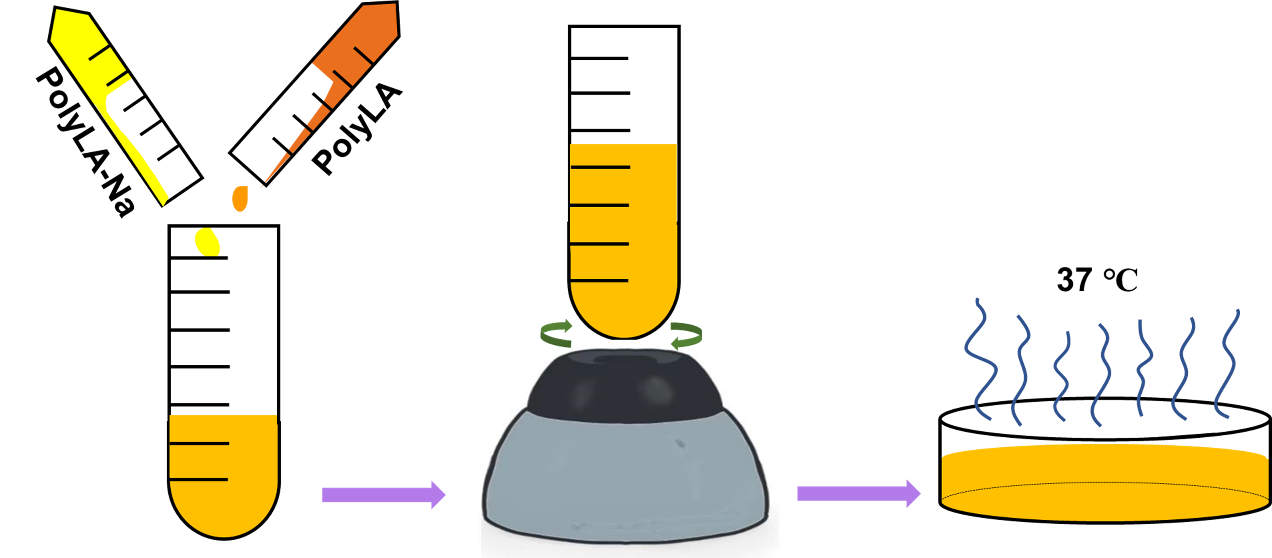


**Supplementary Fig.1 ▏Schematic diagram of the preparation process of PolyLA-Na/PolyLA binary synergistic adhesive patches.**


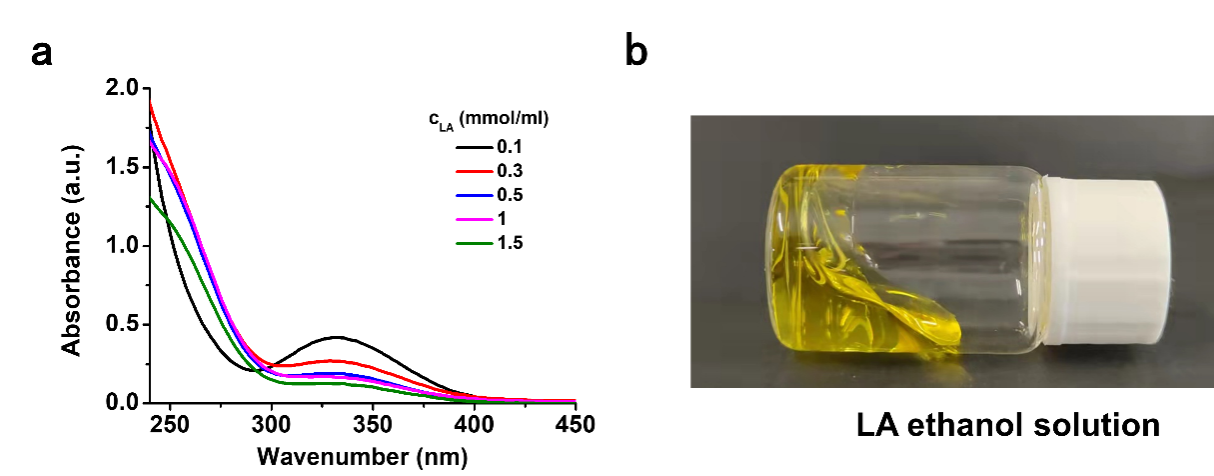


**Supplementary Fig. 2 ▏****Ring-opening spontaneous polymerization of LA in ethanol solution. a:** UV-Vis absorption spectra of LA ethanolic solution with different concentrations; **b:** Photograph of the viscous PolyLA polymer ethanol solution.

**
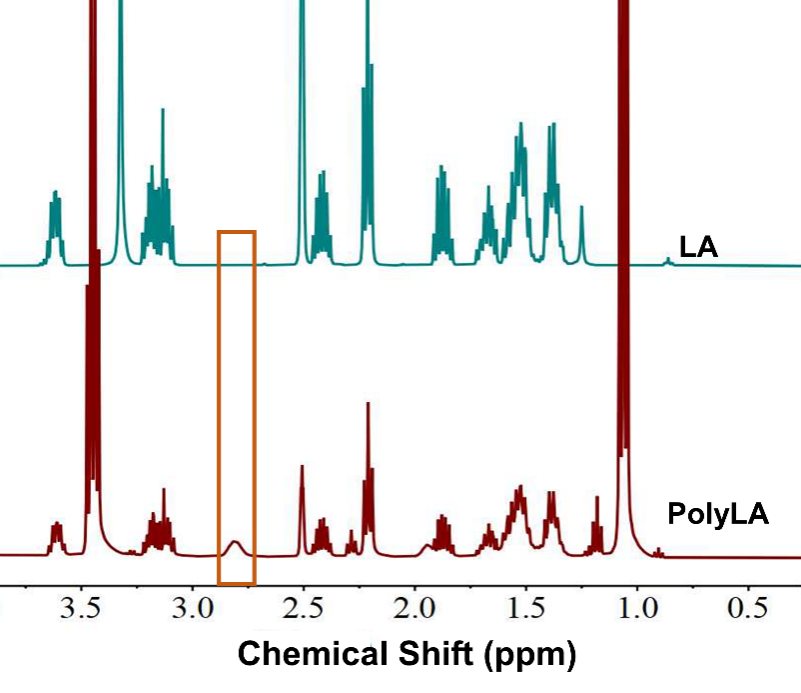
**

**Supplementary Fig. 3 ▏ ^1^H NMR spectra of the LA powder and PolyLA in DMSO-d6.**

**
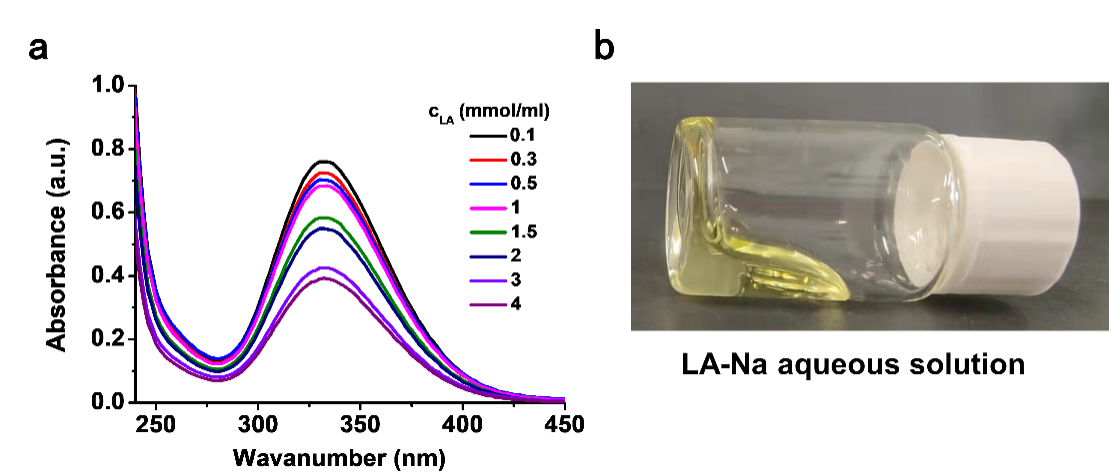
**

**Supplementary Fig. 4 ▏Ring-opening spontaneous polymerization of LA-Na in water solution. a:** UV-Vis absorption spectra of LA-Na aqueous solution with different concentrations; **b:** Photograph of the viscous PolyLA-Na polymer aqueous solution.

**
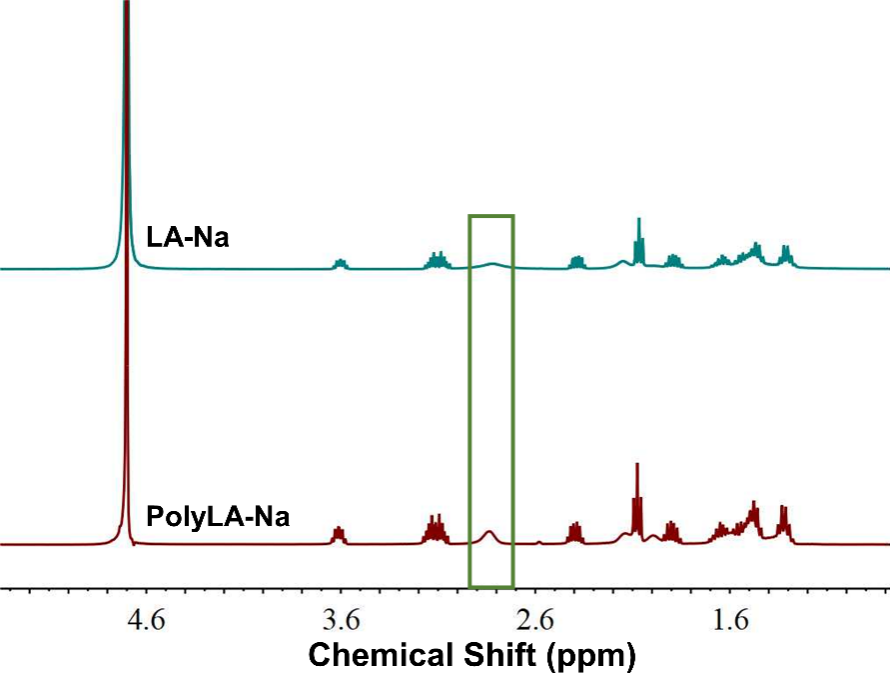
**

**Supplementary Fig. 5 ▏ ^1^H NMR spectra of the LA-Na powder and PolyLA-Na in D_2_O.**

**
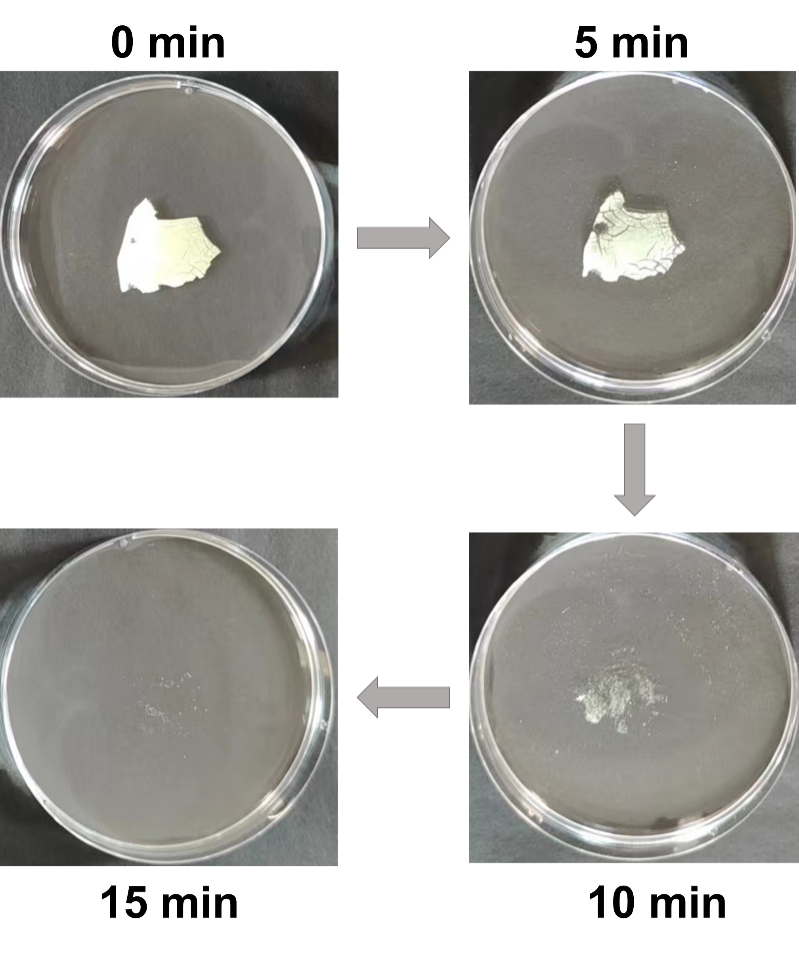
**

**Supplementary Fig. 6 ▏Dissociation process of PolyLA-Na film in water with time at 37 ℃.**

**Supplementary Fig. 7 ▏ FT-IR spectra of the pristine PolyLA-Na film and PolyLA-Na/PolyLA binary synergistic patches with different compositions.**

**Supplementary Fig. 8 ▏XRD patterns of the PolyLA-Na/PolyLA patches with different compositions.**

**
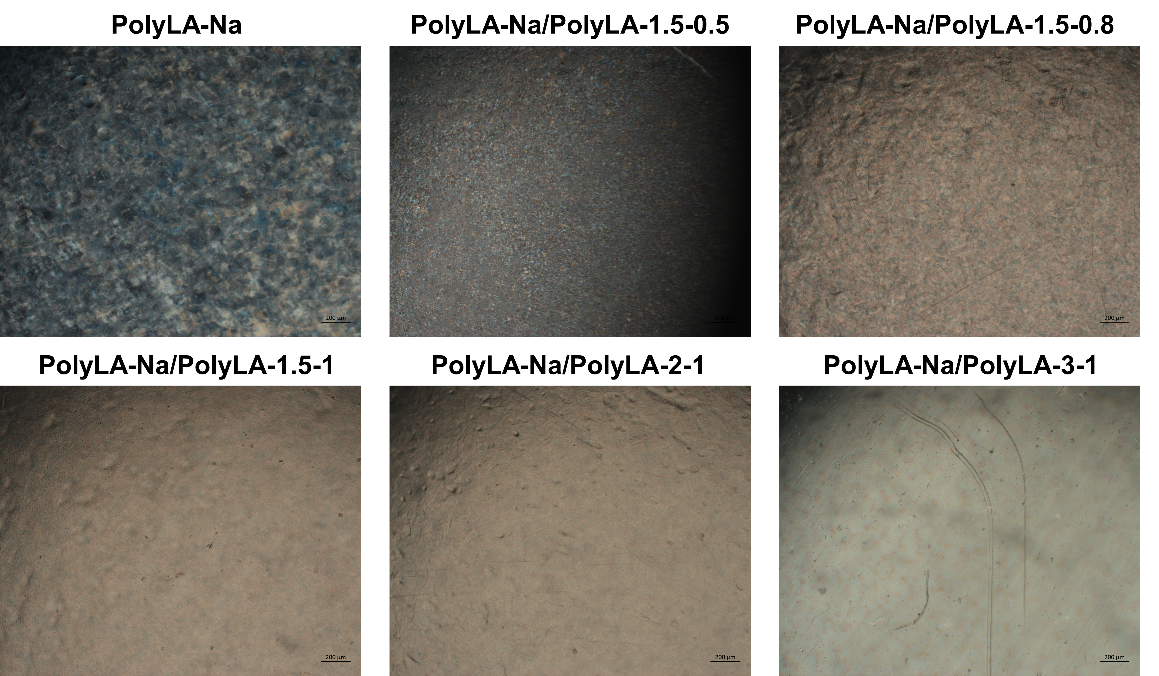
**

**Supplementary Fig. 9 ▏Polarizing microscope pictures of the PolyLA-Na/PolyLA patches with different compositions (n = 1 biologically independent sample in each group).**

**
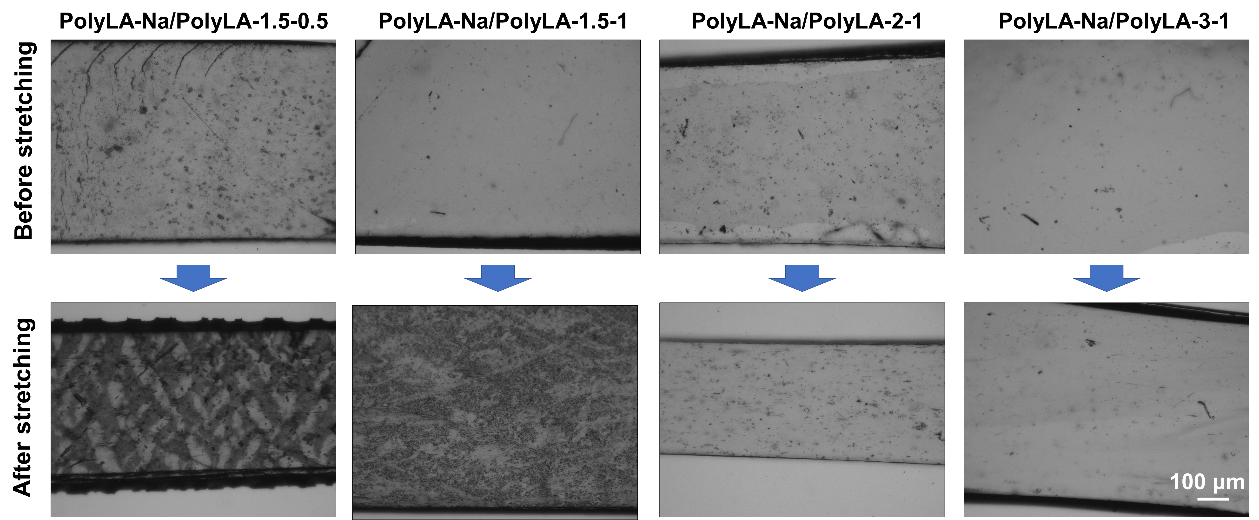
**

**Supplementary Fig. 10 ▏Optical microscope images of the PolyLA-Na/PolyLA patches before and after being stretched (n = 1 biologically independent sample in each group).**

**Supplementary Fig. 11 ▏Crystallinity of the PolyLA-Na/PolyLA patches with different compositions.**

**
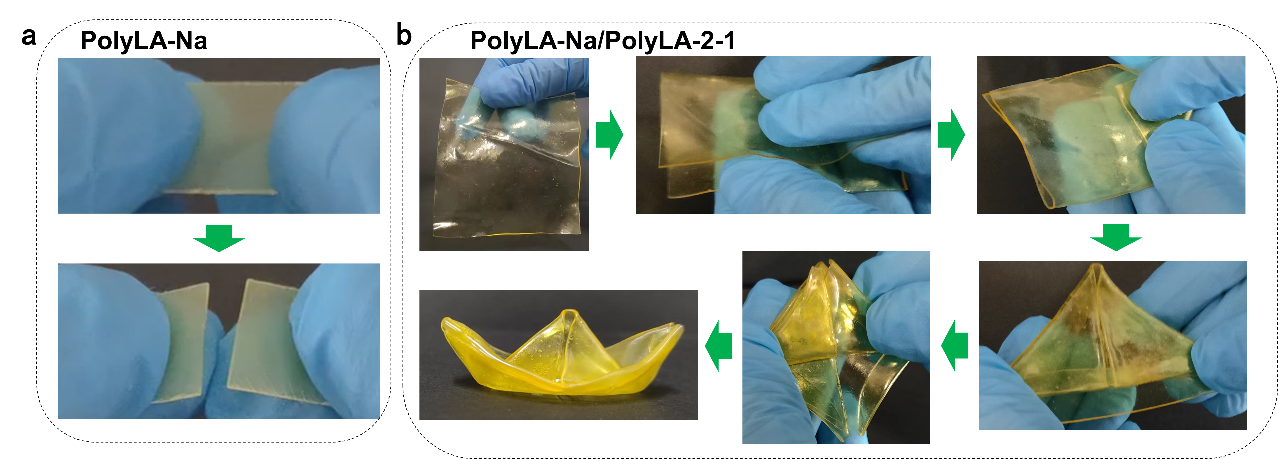
**

**Supplementary Fig. 12 ▏The flexibility comparison of dry PolyLA-Na film and PolyLA-Na/PolyLA patch. a:** Pictures showing the brittle and hard property of the PolyLA-Na film, which broke easily when it was bent; **b:** Pictures showing the soft and flexible properties of the PolyLA-Na/PolyLA-2-1 adhesive patch, which could be folded into complex shapes without breaking.

**
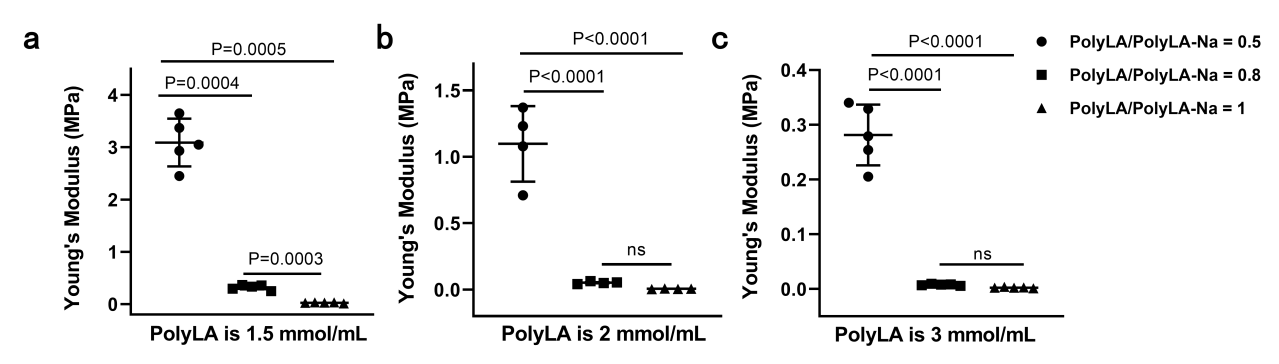
**

**Supplementary Fig. 13 ▏Young’s modulus of PolyLA-Na/PolyLA patches with different compositions.** All presented data are mean values ± SD from the mean from n=4 independent measurements on independent samples. Statistics was calculated by one-way ANOVA followed by Tukey’s post-test.

**
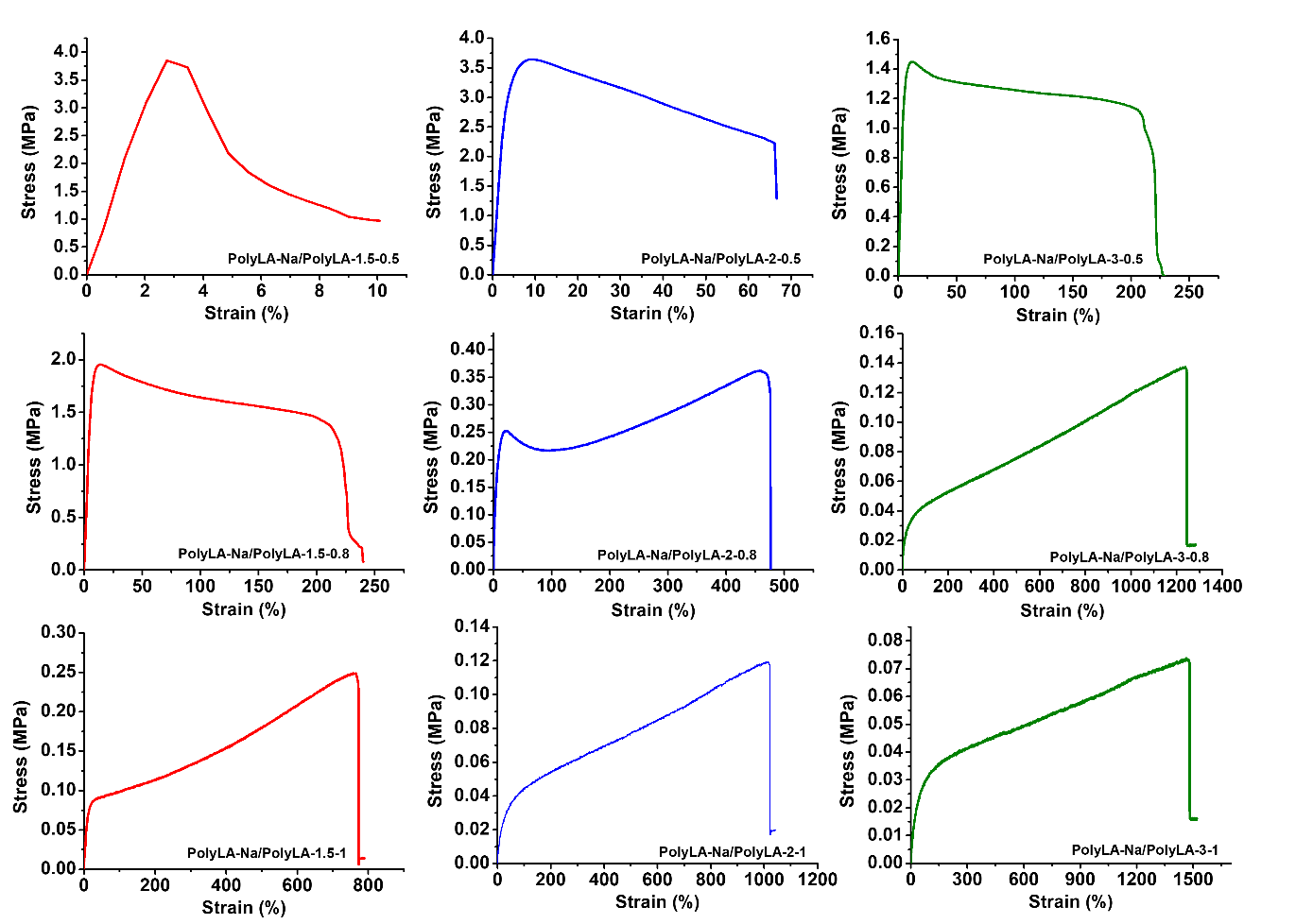
**

**Supplementary Fig. 14 ▏Tensible stress-strain curves of PolyLA-Na/PolyLA patches with different compositions.**

**
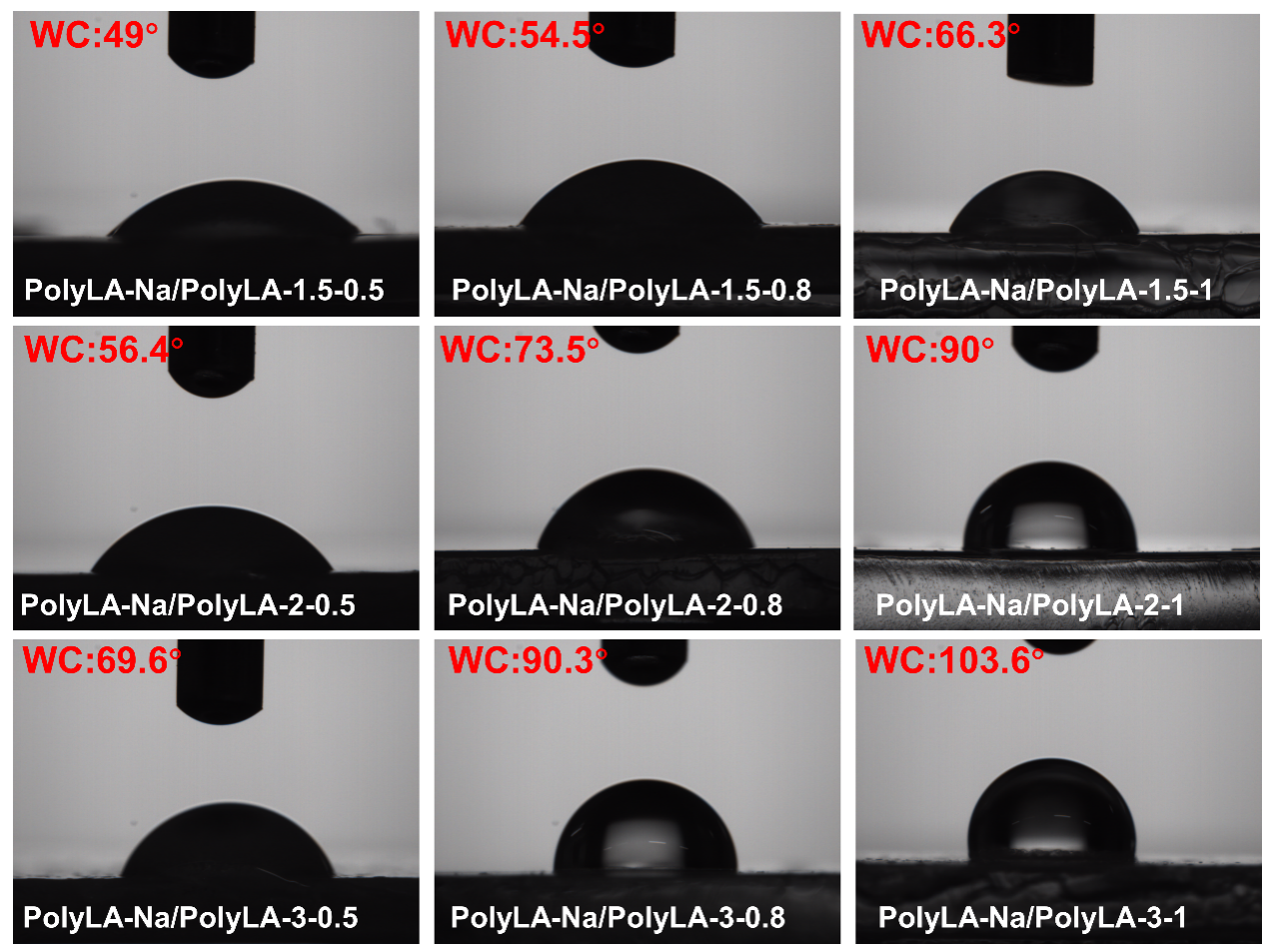
**

**Supplementary Fig. 15 ▏Photographs of water contact angle of PolyLA-Na/PolyLA patches with different compositions after contacting water for 20 s (WC: water contact angle).**

**
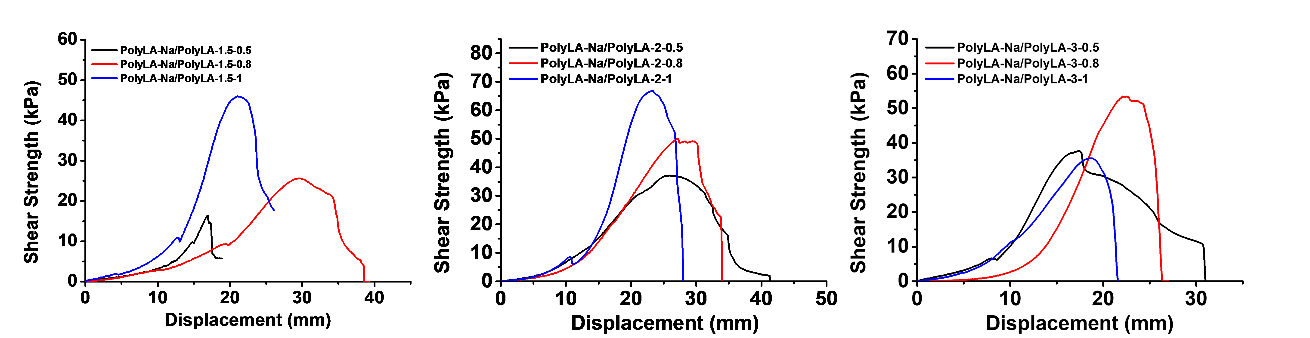
**

**Supplementary Fig. 16 ▏Lap shear adhesion curves of PolyLA-Na/PolyLA patches with different compositions to pig oral mucosa tissue soaked in artificial saliva.**


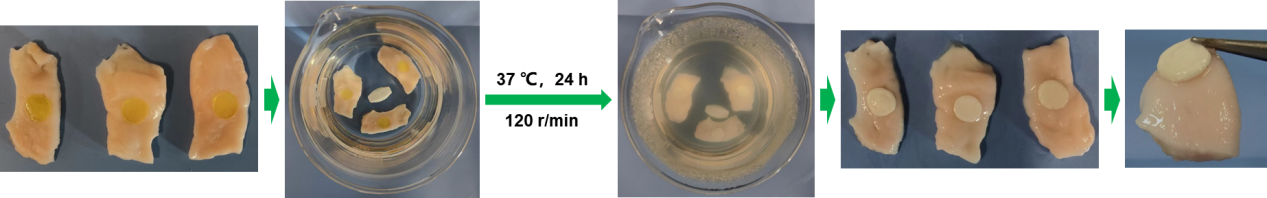


**Supplementary Fig. 17 ▏Durable wet tissue adhesion of PolyLA-Na/PolyLA-2-1 to oral mucosa tissue in the salivary environment at 37 ^°^C.**

**Supplementary Fig. 18 ▏Swelling behavior of PolyLA-Na/PolyLA adhesive patches with different compositions in artificial saliva (n=3).**

**
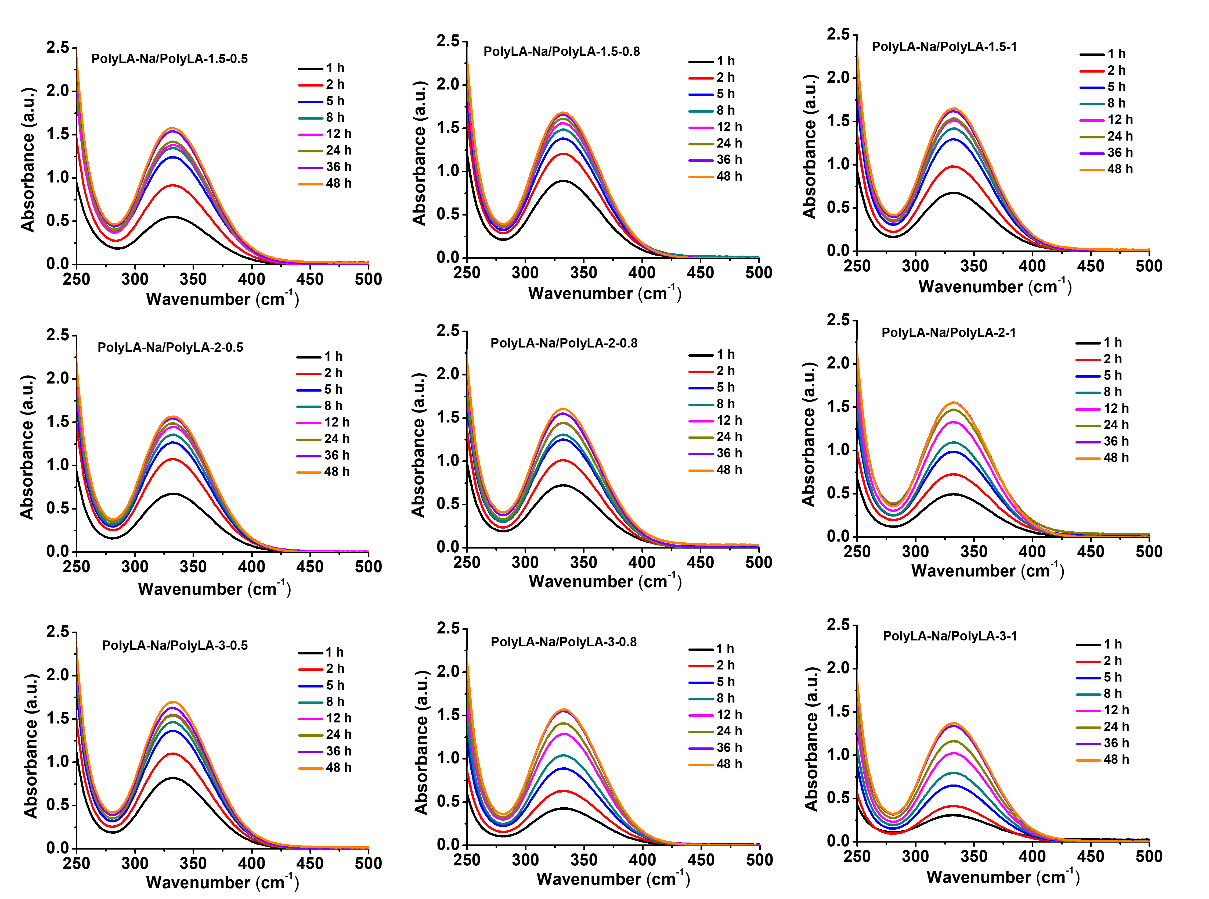
**

**Supplementary Fig. 19 ▏UV-Vis spectra of monomer release from PolyLA-Na/PolyLA patches with different compositions.**

**Supplementary Fig. 20 ▏Monomer release ratio of PolyLA-Na/PolyLA adhesive patches with different compositions in artificial saliva for different times (n=3).**

**Supplementary Fig. 21 ▏DPPH radical clearance ratio of PolyLA-Na/PolyLA adhesive patches determined at different times (n=3).**

**
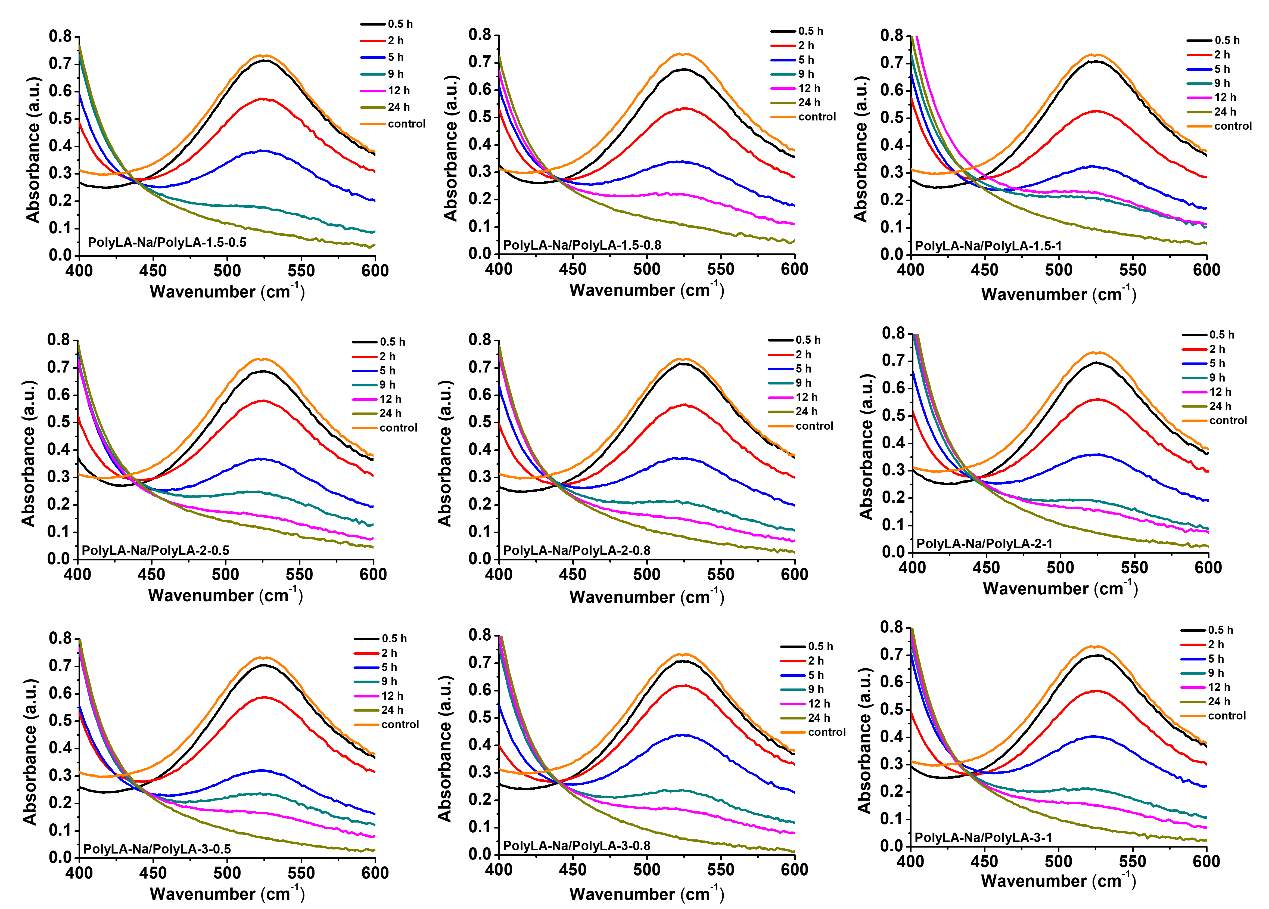
**

**Supplementary Fig. 22 ▏Absorbance curves of DPPH solution treated with PolyLA-Na/PolyLA adhesive patches at different times.**


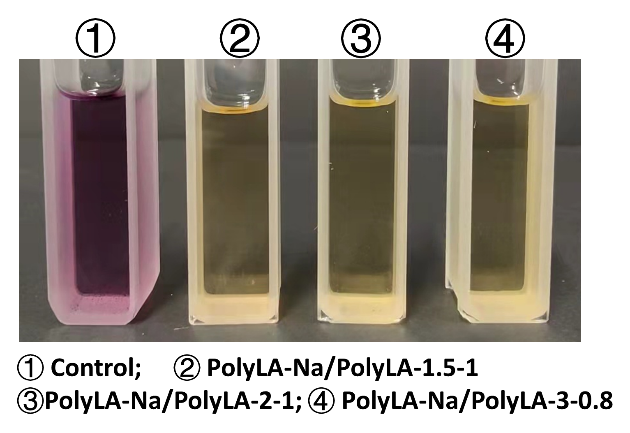


**Supplementary Fig. 23 ▏Photographs of DPPH solutions in the absence of (control) and presence of DPPH/PolyLA-Na/PolyLA adhesive patches after treatment for 24 h.**


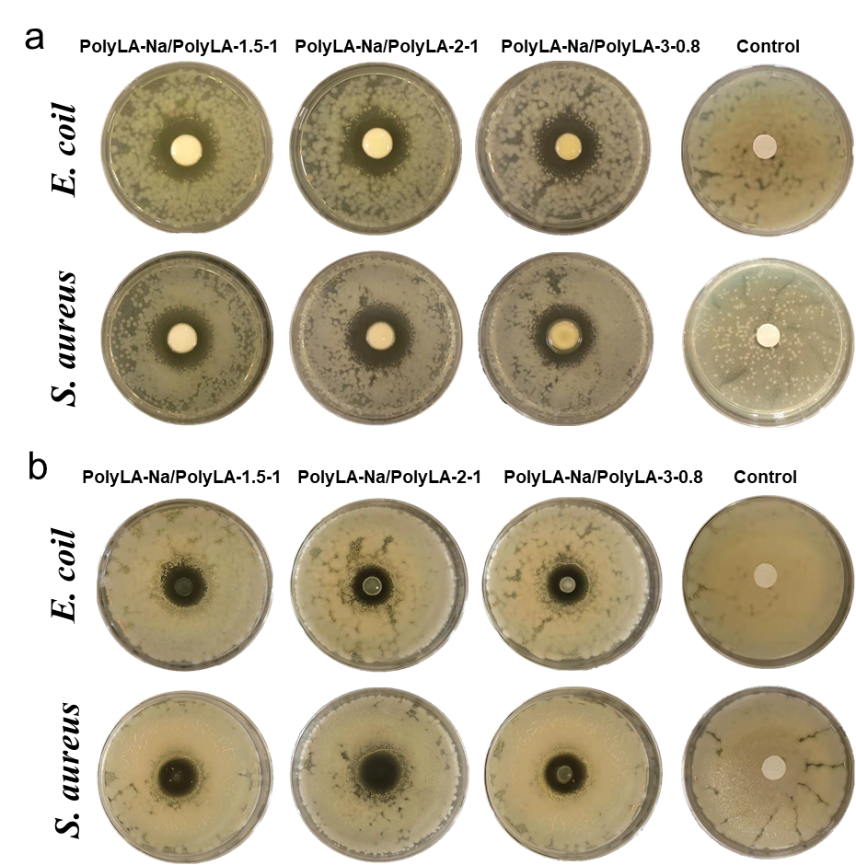


**Supplementary Fig. 24 ▏Digital images displaying representative inhibition zone results for the PolyLA-Na/PolyLA adhesive patches against *E.coli* and *S.aureus* at 12 (a) and 24 h (b).**


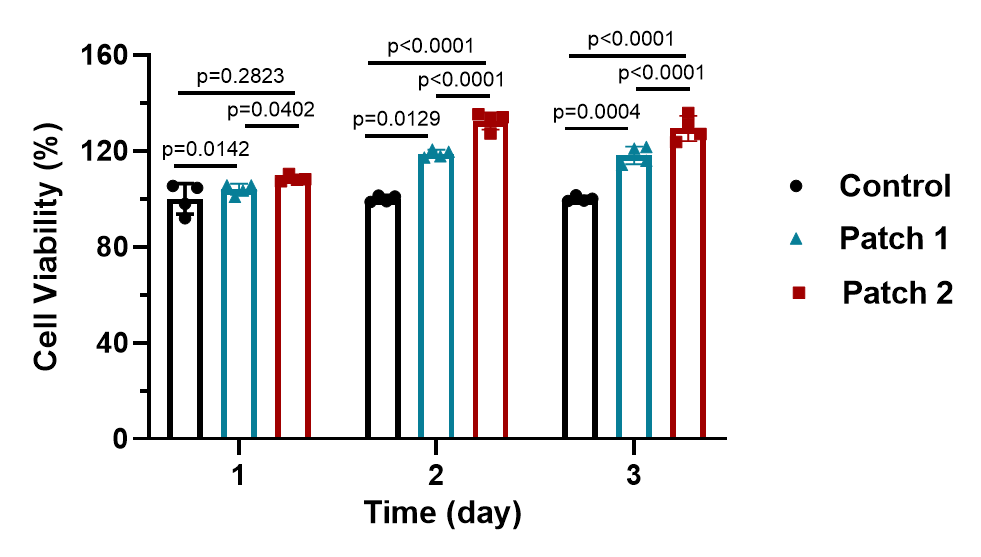


**Supplementary Fig. 25 ▏Cell viability of** **HUVECs cells after co-cultured with PolyLA-Na/PolyLA patch for 1, 2, 3 days.** All presented data are mean values ± SD from the mean from n=4 independent measurements on independent samples. All p-values were calculated using a two-sided Student’s t-test.


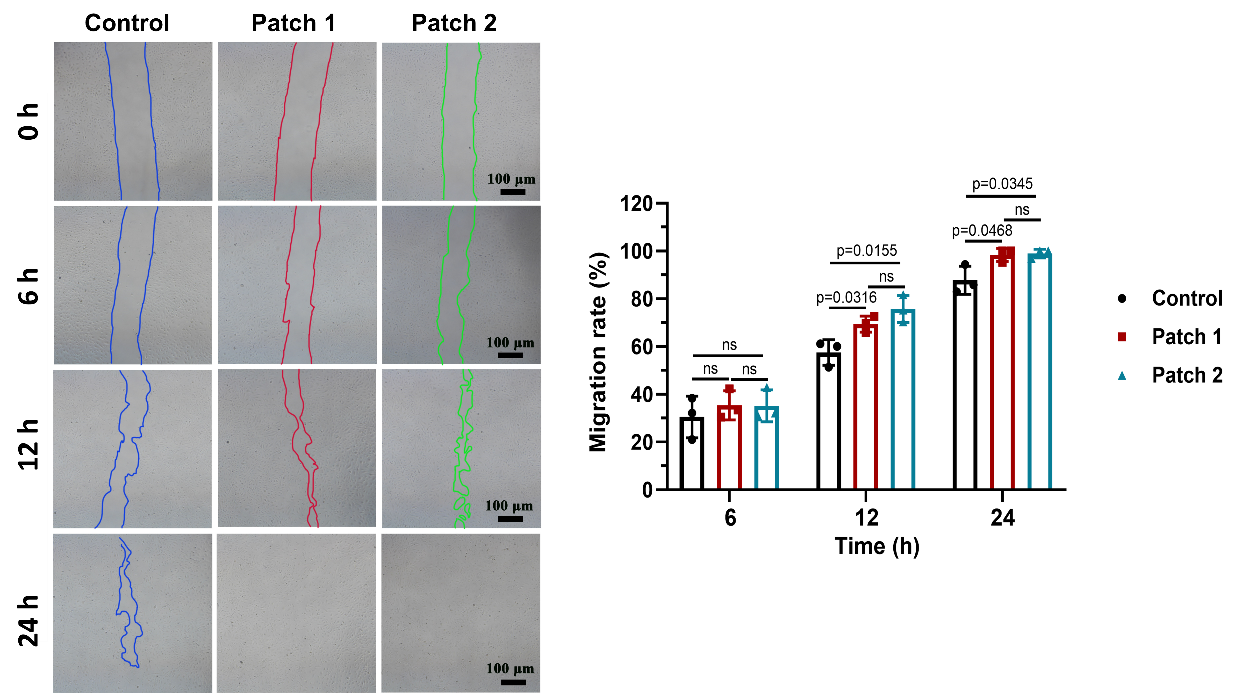


**Supplementary Fig. 26 ▏Representative images of** **HUVECs cell migration at different times and the migration rate of HUVECs cells co-cultured with PolyLA-Na/PolyLA patch for different time.** All presented data are mean values ± SD from the mean from n=3 independent measurements on independent samples. All p-values were calculated using a two-sided Student’s t-test.


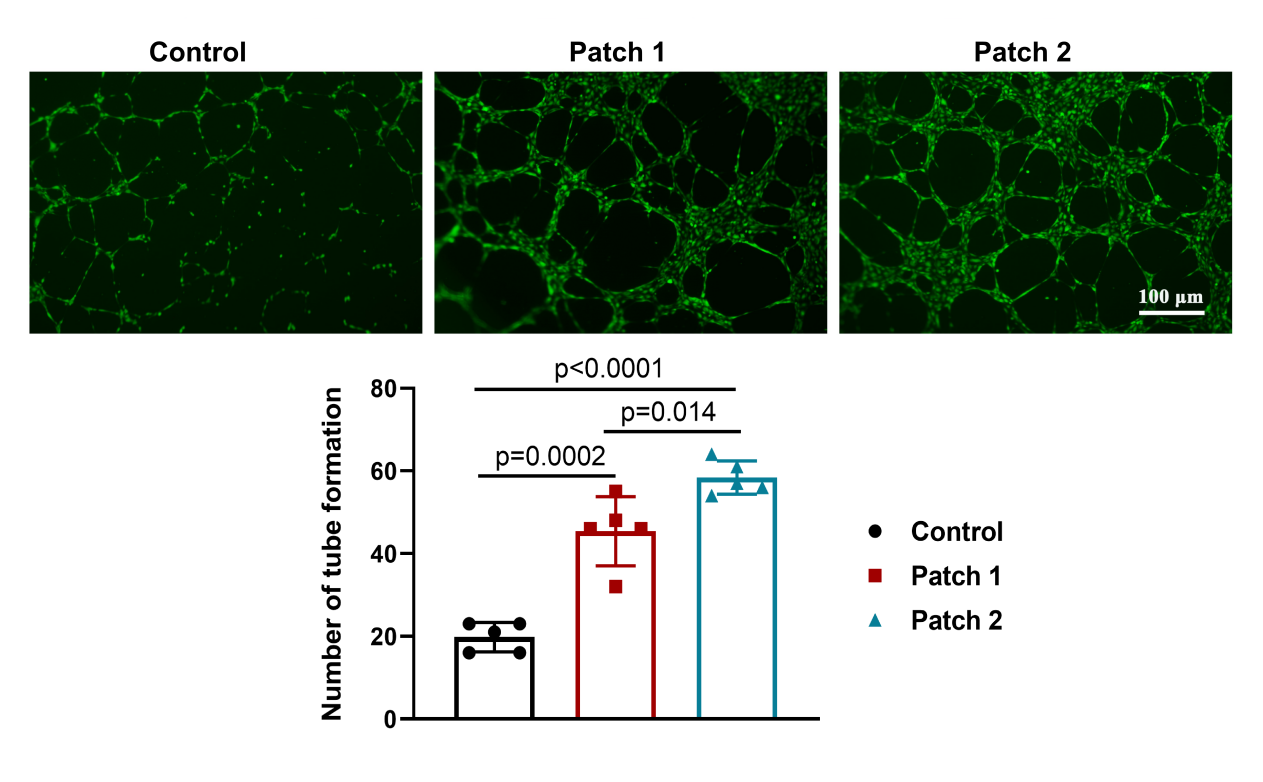


**Supplementary Fig. 27 ▏Representative images of capillary-like structures of HUVECs and** **quantification of formed tube number of HUVECs cells co-cultured with PolyLA-Na/PolyLA patch.** All presented data are mean values ± SD from the mean from n=5 independent measurements on independent samples. All p-values were calculated using a two-sided Student’s t-test.


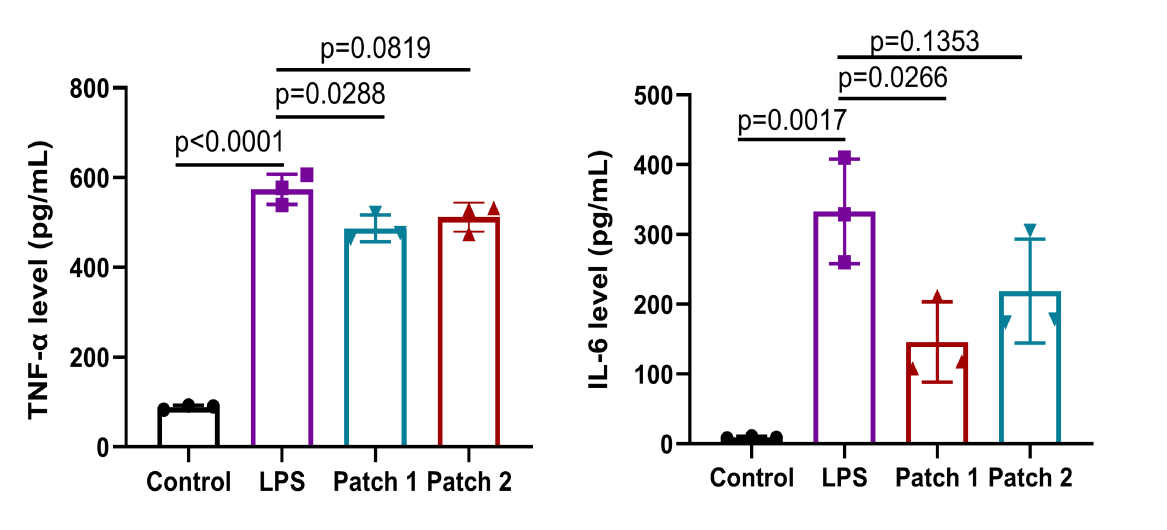


**Supplementary Fig. 28 ▏Effect of PolyLA-Na/PolyLA patch on TNF-α and IL-6 production in LPS stimulated RAW264.7 cells.** All presented data are mean values ± SD from the mean from n=3 independent measurements on independent samples. All p-values were calculated using a two-sided Student’s t-test.


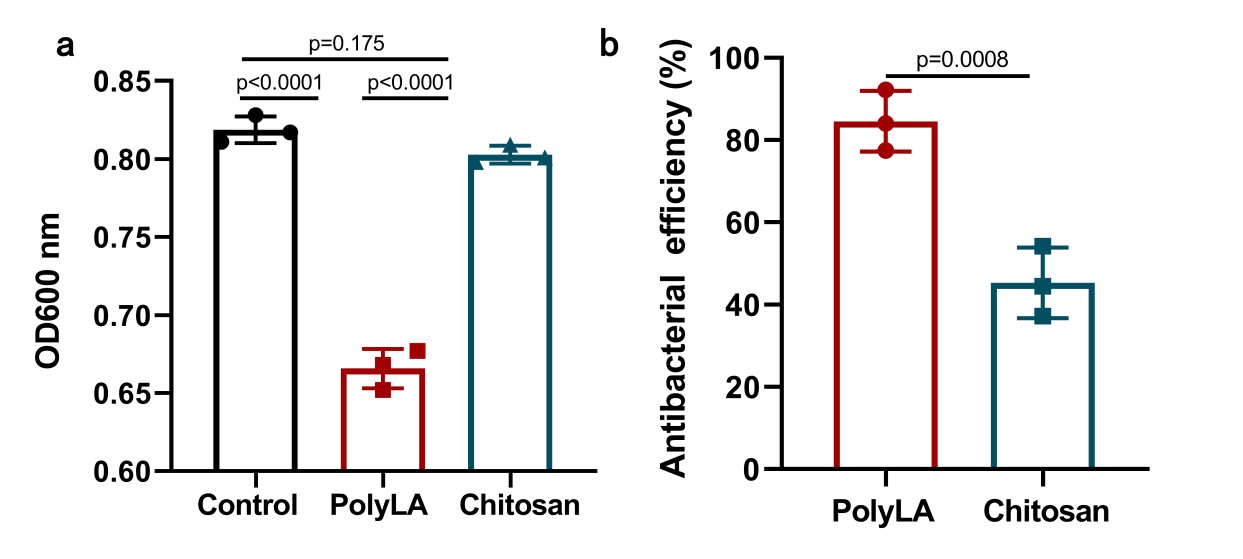


**Supplementary Fig. 29 ▏Antibacterial effect of PolyLA-Na/PolyLA-2-1 patch in oral cavity. a:** OD_600_ nm values of control group, PolyLA-Na/PolyLA-2-1 patch group and chitosan film group after 12 hours of bacterial culture; **b:** Antibacterial efficiency of PolyLA-Na/PolyLA-2-1 patch group and chitosan film group in vivo. All presented data are mean values ± SD from the mean from n=3 independent measurements on independent samples. All p-values were calculated using a two-sided Student’s t-test.


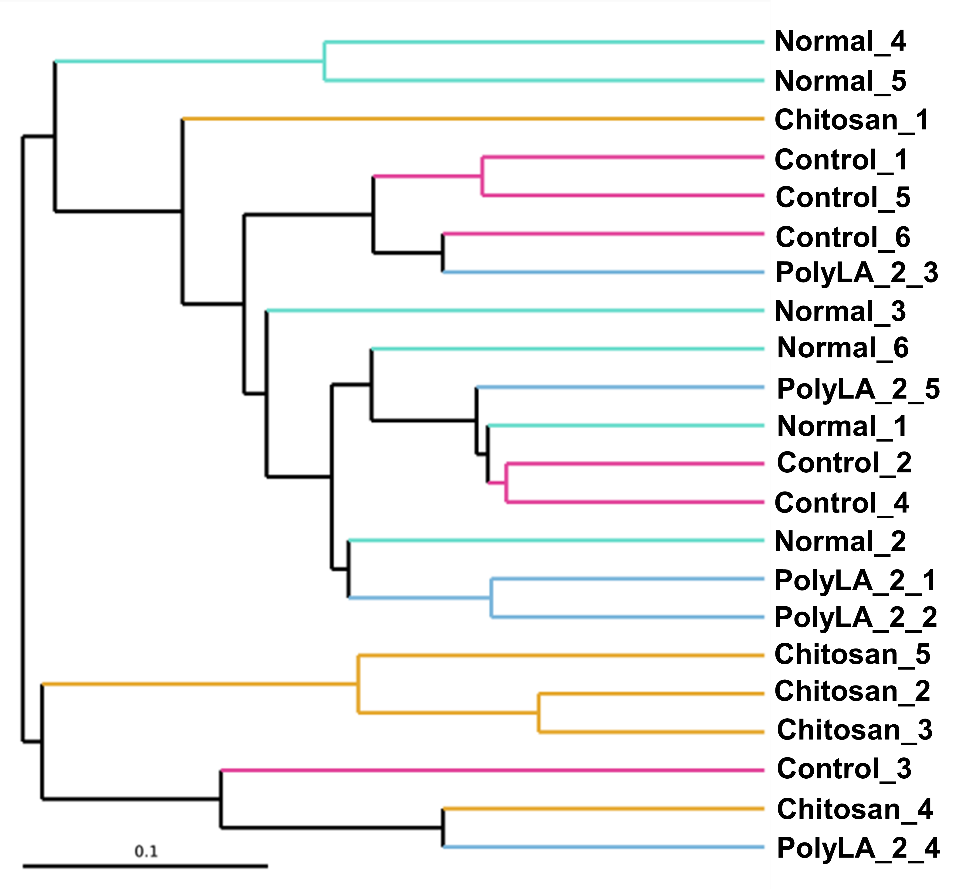


**Supplementary Fig. 30 ▏Analysis of bacterial clusters in individual samples based on the ASVs.**


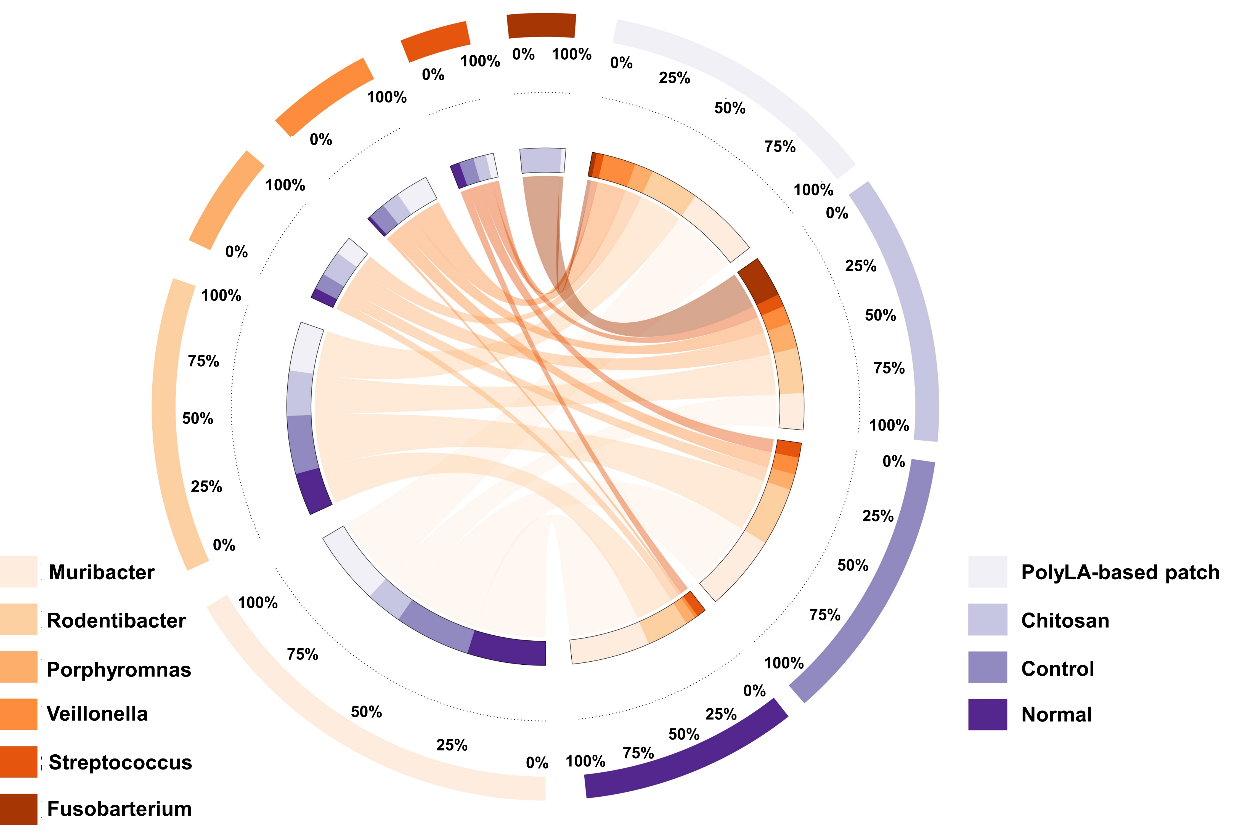


**Supplementary Fig. 31 ▏Bacterial species of different groups at the genus level.**


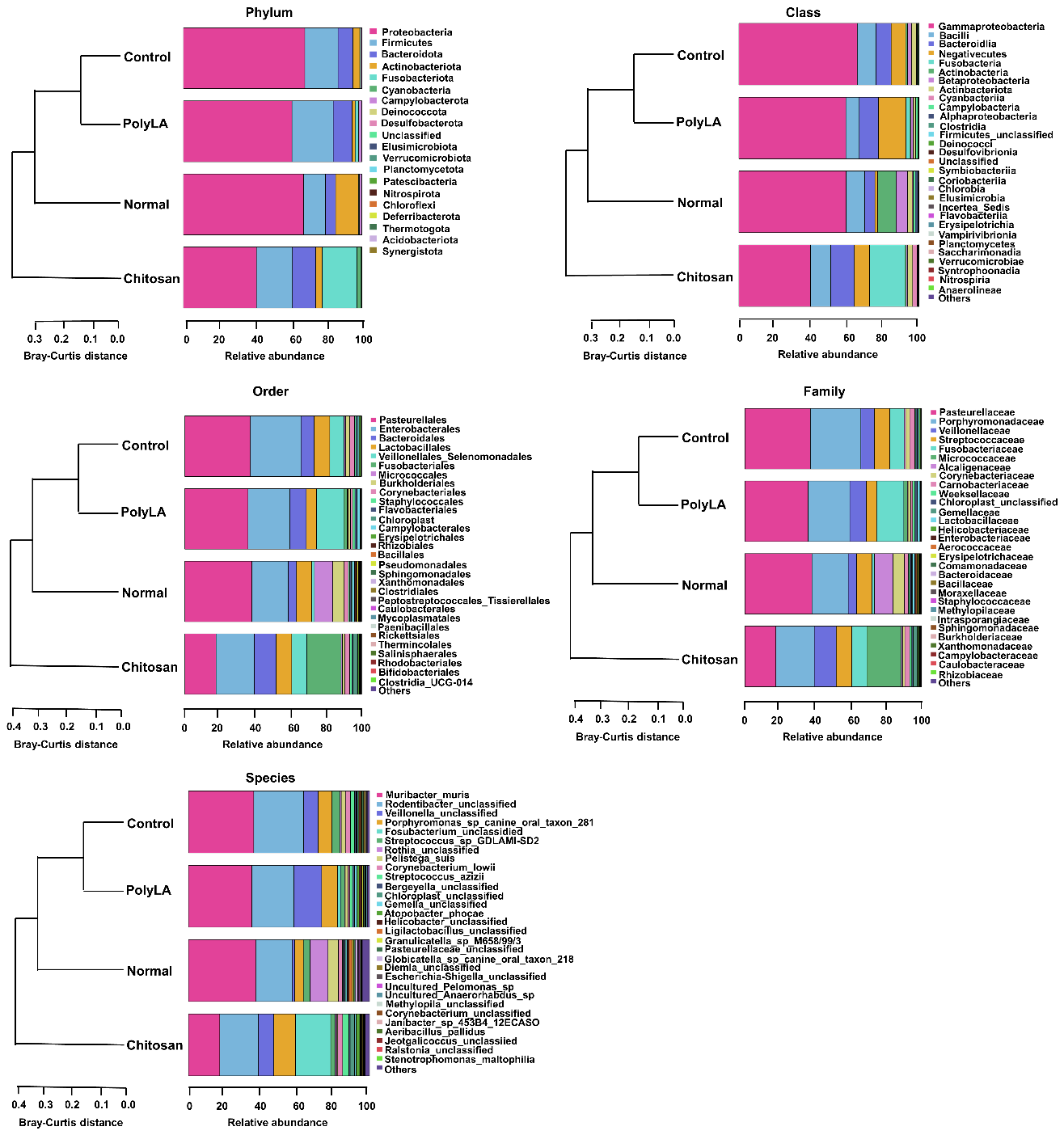


**Supplementary Fig. 32 ▏Bacteria cluster analysis of different groups at the level of phylum, class, order, family, and species.**


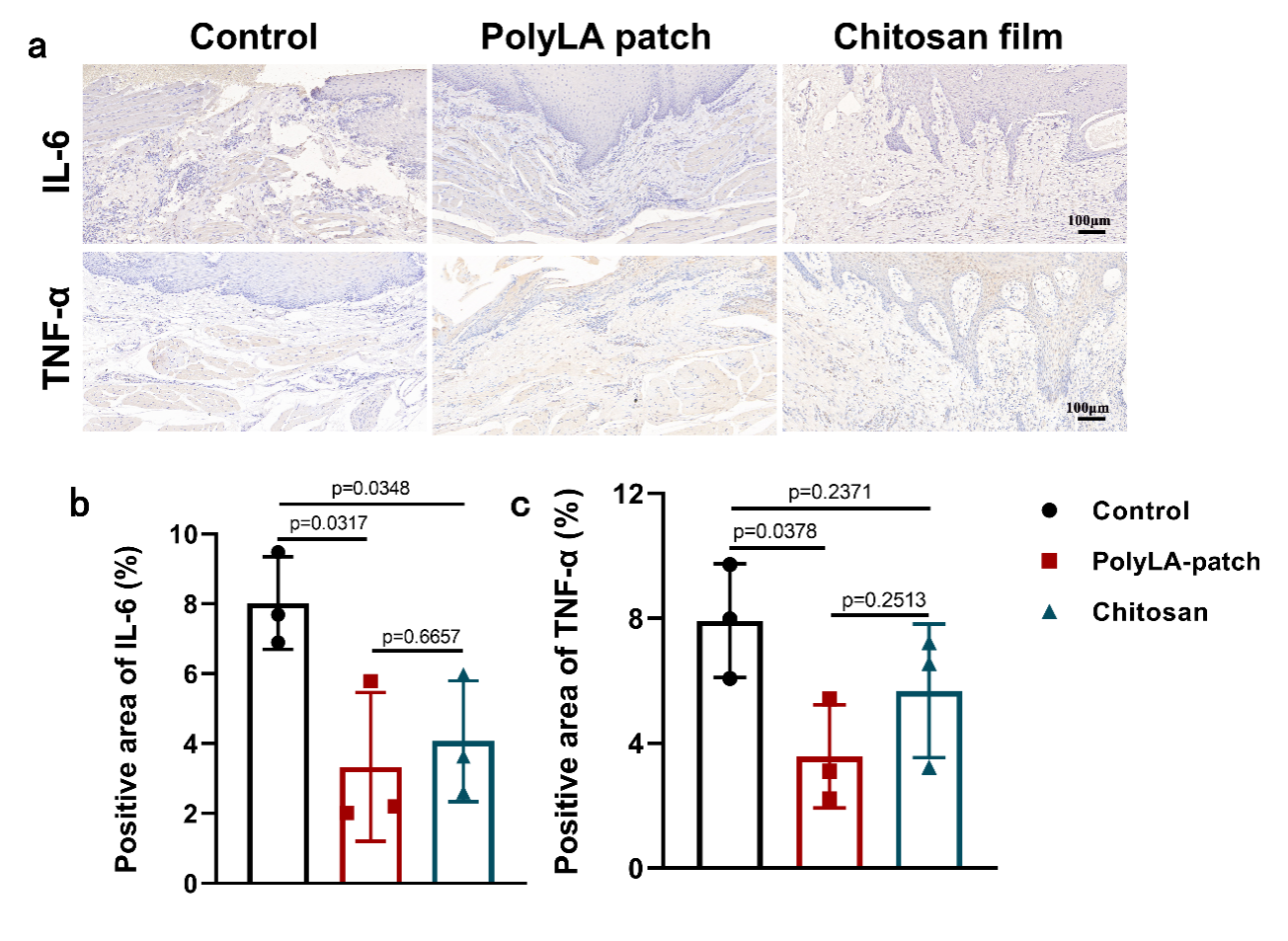


**Supplementary Fig. 33 ▏Expression of anti-inflammatory factors in different groups. a:** Immunohistochemical staining of IL-6 and TNF-α antibody in regenerated oral mucosa at 8 days; **b-c:** Quantification of expression levels of IL-6 and TNF-α in regenerated oral mucosa. All presented data are mean values ± SD from the mean from n=3 independent measurements on independent samples. All p-values were calculated using a two-sided Student’s t-test.

**Supplementary Table 1**. *In vivo* plasma concentration of LA-based molecule at different times after attachment of PolyLA-Na/Poly LA on the *rat* buccal mucosa.

| Time | Rat 1 | Rat 2 | Rat 3 |
| --- | --- | --- | --- |
| 6 h | 0.926 | 0.259 | 0.241 |
| 12 h | <0.2 | 0.288 | <0.2 |
| 24 h | <0.2 | <0.2 | <0.2 |

**Supplementary Table 2**. Comparison of PolyLA-Na/PolyLA-based patch with previous works (/ indicates parameters that were not mentioned in previous work).

| Materials | Load component | Adhesion time in vivo | Adhesion strength/mode |
| --- | --- | --- | --- |
| PolyLA-Na/PolyLA based patch | Without any exogenous components | > 12 h | 60 kPa/slight pressing for 5 s |
| 3D printing PAA-DOPA-CMC patch^[1]^ | Oxaliplatin/Mycophenolate | 4 h | 1.29 ± 0.30 N/5 N pressing |
| Chitosan-HA patch^[2]^ | Ovalbumin | / | / |
| PVP-PEP bilayer electrospun patch^[3]^ | Clobetasol-17-propionate | 4 h | / |
| PVP-Eudragit electrospun patch^[4]^ | anti-TNFα | 3 h | / |
| PCL-PLA-PEO-multilayer electrospun patch^[5]^ | Ciprofloxacin hydrochloride | 7 h | / |
| PCL-Gelatin-PGA electrospun patch^[6]^ | Astaxanthin | 2 h | / |
| HA-CNB Gel^[7]^ | / | 24 h | 70 kPa/UV illumination |
| Gelatin-PDA-nanoclay hydrogel^[8]^ | Dexamethasone | / | 63 kPa/pressing for 30 s |
| PVA-DOPA film^[9]^ | Dexamethasone | > 4 h | 38.72 ± 10.94 kPa |
| GelMA-TA-nanoclay hydrogel^[10]^ | / | 10 h | 20 kPa |
| PACG-MeGG hydrogel^[11]^ | / | 4 h | 3 kPa |
| PAA-chitosan dry gel^[12]^ | 5-aminolevulinic acid | / | 40 kPa |

**Supplementary References:**

1. Liu, X. Q. et al. Designing a mucoadhesive chemoPatch to ablate oral dysplasia for cancer prevention. *Small* **18**, 2201561(2022).

2. Paris, A. L. et al. Sublingual protein delivery by a mucoadhesive patch made of natural polymers. *Acta Biomater.* **128**, 222-235(2021).

3. Colley, H. E. et al. Pre-clinical evaluation of novel mucoadhesive bilayer patches for local delivery of clobetasol-17-propionate to the oral mucosa. *Biomaterials* **178**, 134-146(2018).

4. Edmans, J. G. et al. Electrospun patch delivery of anti-TNFα F (ab) for the treatment of inflammatory oral mucosal disease. *J. Control. Release* **350**, 146-157(2022).

5. Teno, J. et al. Development of multilayer ciprofloxacin hydrochloride electrospun patches for buccal drug delivery. *J. Funct. Biomater.* **13**, 170(2022).

6. Zhang, H. et al. Fabrication of astaxanthin-loaded electrospun nanofiber-based mucoadhesive patches with water-insoluble backing for the treatment of oral premalignant lesions. *Mater. Design* **223**, 111131(2022).

7. Zhang, W. J. et al. Promoting Oral mucosal wound healing with a hydrogel adhesive based on a phototriggered s-nitrosylation coupling reaction. *Adv. Mater.* **33**, 2105667(2021).

8. An, H. et al. Janus mucosal dressing with a tough and adhesive hydrogel based on synergistic effects of gelatin, polydopamine, and nano-clay. *Acta Biomater.* **149**, 126-138(2022).

9. Hu, S. S. et al. A mussel-inspired film for adhesion to wet buccal tissue and efficient buccal drug delivery. *Nat. Commun.* **12**, 1689(2021).

10. Zhu, J. J. et al. Low-swelling adhesive hydrogel with rapid hemostasis and potent anti-Inflammatory capability for full-Thickness oral mucosal defect Rrepair. *ACS Appl. Mater. Interfaces* **14**, 53575-53592(2022).

11. Xing, J. Q. et al. Barnacle-Inspired robust and aesthetic Janus patch with instinctive wet adhesive for oral ulcer treatment. *Chem. Eng. J.* **444**, 136580(2022).

12. Wang, X. et al. Hydrogel-based patient-friendly photodynamic therapy of oral potentially malignant disorders. *Biomaterials* **281**, 121377(2022).
