# Supplementary material for: A self-stabilized and water-responsive deliverable coenzyme-based polymer binary elastomer adhesive patch for treating oral ulcer
Source: Nat Commun. 2023 Nov 24;14:7707. doi: 10.1038/s41467-023-43571-x (PMC10673908; doi:10.1038/s41467-023-43571-x)
Supplement: Supplementary file 2 — Reporting Summary [file 41467_2023_43571_MOESM2_ESM.pdf]

nature portfolio

Corresponding author(s): Wenguang Liu

Last updated by author(s): Sep 13, 2023

Reporting Summary

Nature Portfolio wishes to improve the reproducibility of the work that we publish. This form provides structure for consistency and transparency in reporting. For further information on Nature Portfolio policies, see our Editorial Policies and the Editorial Policy Checklist.

Statistics

For all statistical analyses, confirm that the following items are present in the figure legend, table legend, main text, or Methods section.

n/a	Confirmed	
☐	☒	The exact sample size (*n*) for each experimental group/condition, given as a discrete number and unit of measurement
☐	☒	A statement on whether measurements were taken from distinct samples or whether the same sample was measured repeatedly
☐	☒	The statistical test(s) used AND whether they are one- or two-sided<br>*Only common tests should be described solely by name; describe more complex techniques in the Methods section.*
☐	☒	A description of all covariates tested
☒	☐	A description of any assumptions or corrections, such as tests of normality and adjustment for multiple comparisons
☐	☒	A full description of the statistical parameters including central tendency (e.g. means) or other basic estimates (e.g. regression coefficient) AND variation (e.g. standard deviation) or associated estimates of uncertainty (e.g. confidence intervals)
☒	☐	For null hypothesis testing, the test statistic (e.g. *F*, *t*, *r*) with confidence intervals, effect sizes, degrees of freedom and *P* value noted<br>*Give P values as exact values whenever suitable.*
☒	☐	For Bayesian analysis, information on the choice of priors and Markov chain Monte Carlo settings
☒	☐	For hierarchical and complex designs, identification of the appropriate level for tests and full reporting of outcomes
☒	☐	Estimates of effect sizes (e.g. Cohen's *d*, Pearson's *r*), indicating how they were calculated

Our web collection on statistics for biologists contains articles on many of the points above.

Software and code

Policy information about availability of computer code

Data collection	Office 2016, Inverted fluorescence microscopy (Nikon A1 MP, Japan), Microplate reader (Synergy H1/H1M, Bio-Tek, China)
Data analysis	rigin 8.0, Origin 9.0, Prism 8.0, ChemDraw 20.0, Image J

For manuscripts utilizing custom algorithms or software that are central to the research but not yet described in published literature, software must be made available to editors and reviewers. We strongly encourage code deposition in a community repository (e.g. GitHub). See the Nature Portfolio guidelines for submitting code & software for further information.

Data

Policy information about availability of data

All manuscripts must include a data availability statement. This statement should provide the following information, where applicable:

- Accession codes, unique identifiers, or web links for publicly available datasets
- A description of any restrictions on data availability
- For clinical datasets or third party data, please ensure that the statement adheres to our policy

The data generated in this study are provided in the Supplementary Information/Source Data file. The full image dataset is available from the corresponding author upon request.

Research involving human participants, their data, or biological material

Policy information about studies with human participants or human data. See also policy information about sex, gender (identity/presentation), and sexual orientation and race, ethnicity and racism.

Reporting on sex and gender	*Use the terms sex (biological attribute) and gender (shaped by social and cultural circumstances) carefully in order to avoid confusing both terms. Indicate if findings apply to only one sex or gender; describe whether sex and gender were considered in study design; whether sex and/or gender was determined based on self-reporting or assigned and methods used. Provide in the source data disaggregated sex and gender data, where this information has been collected, and if consent has been obtained for sharing of individual-level data; provide overall numbers in this Reporting Summary. Please state if this information has not been collected. Report sex- and gender-based analyses where performed, justify reasons for lack of sex- and gender-based analysis.*
Reporting on race, ethnicity, or other socially relevant groupings	*Please specify the socially constructed or socially relevant categorization variable(s) used in your manuscript and explain why they were used. Please note that such variables should not be used as proxies for other socially constructed/relevant variables (for example, race or ethnicity should not be used as a proxy for socioeconomic status). Provide clear definitions of the relevant terms used, how they were provided (by the participants/respondents, the researchers, or third parties), and the method(s) used to classify people into the different categories (e.g. self-report, census or administrative data, social media data, etc.) Please provide details about how you controlled for confounding variables in your analyses.*
Population characteristics	*Describe the covariate-relevant population characteristics of the human research participants (e.g. age, genotypic information, past and current diagnosis and treatment categories). If you filled out the behavioural & social sciences study design questions and have nothing to add here, write "See above."*
Recruitment	*Describe how participants were recruited. Outline any potential self-selection bias or other biases that may be present and how these are likely to impact results.*
Ethics oversight	*Identify the organization(s) that approved the study protocol.*

Note that full information on the approval of the study protocol must also be provided in the manuscript.

Field-specific reporting

Please select the one below that is the best fit for your research. If you are not sure, read the appropriate sections before making your selection.

☒ Life sciences ☐ Behavioural & social sciences ☐ Ecological, evolutionary & environmental sciences

For a reference copy of the document with all sections, see nature.com/documents/nr-reporting-summary-flat.pdf

Life sciences study design

All studies must disclose on these points even when the disclosure is negative.

Sample size	Ex vivo experiments on porcine tissues were conducted to evaluate adhesion performance of the PolyTA-based adhesive. The appropriate sample size (n=3-5) was used for each test. In the in vivo experiment of rats and pigs, the ability of PolyTA-based adhesive patch to promote oral tissue repair was investigated, and the appropriate sample size (n=3 for rat and n=5 for pig) was used to evaluate its effect in vivo.
Data exclusions	Data with too high or too low values are excluded for ex vivo experiments.<br>No data was excluded for vivo experiments
Replication	Ex vivo studies for mechanical and adhesion characterization of the PolyTA-based elastomer were reliably reproduced. The average and standard deviation were reported for each test. n = 5 independent samples in each group.<br>Ex vivo studies for cell cell activity of the PolyLA-based elastomer were reliable reproduced. n = 4 biologically independent samples in each group.<br>In vivo studies for biocompatibility, anti-inflammation and promoting repair were reliably reproduced based on similar histological assessment for each case by the blinded pathologist, n = 3 biologically independent samples in each group. Mechanical and adhesion performance was compromised when sample was defective due to expired chemical.
Randomization	The distribution of animals in each group was random, but the order of modeling in each group was not randomly set. In this work, we first model the PolyLA-based elastomer patch group, then the chitosan group, and finally the control group. Since all models are completed on the same day, the order of modeling should not affect the results.
Blinding	All histological assessments were conducted by the blinded pathologist without informing type or group of samples. Other experimental results are obtained from intuitive data obtained by testing, and the author is not affected by grouping when analyzing the data.

Reporting for specific materials, systems and methods

We require information from authors about some types of materials, experimental systems and methods used in many studies. Here, indicate whether each material, system or method listed is relevant to your study. If you are not sure if a list item applies to your research, read the appropriate section before selecting a response.

Materials & experimental systems

n/a	Involved in the study	
☒	☐	Antibodies
☐	☒	Eukaryotic cell lines
☒	☐	Palaeontology and archaeology
☐	☒	Animals and other organisms
☒	☐	Clinical data
☒	☐	Dual use research of concern
☒	☐	Plants

Methods

n/a	Involved in the study	
☒	☐	ChIP-seq
☒	☐	Flow cytometry
☒	☐	MRI-based neuroimaging

Eukaryotic cell lines

Policy information about cell lines and Sex and Gender in Research

Cell line source(s)	RAW264.7 cells (CL-0190), purchased from Procell Life Science&Technology Co., Ltd. RAW 264.7 cells were derived from Abelson murine leukemia virus-induced tumors of adult male mice. HUVECs are SV40T transforms human umbilical vein endothelial cells, iCell-h110, purchased from iCell Bioscience Inc,Shanghai.
Authentication	HUVECs were identified by immunofluorescence and RAW264.7 cells were identified by STR.
Mycoplasma contamination	The cell lines were not contaminated with mycoplasma.
Commonly misidentified lines (See ICLAC register)	Not involved

Animals and other research organisms

Policy information about studies involving animals; ARRIVE guidelines recommended for reporting animal research, and Sex and Gender in Research

Laboratory animals	Sprague Dawley (SD) rats, (male, 8-week-old) and Bama fragrant pig （female, 6-month-old）
Wild animals	This study did not involve wild animals
Reporting on sex	This study is applicable to all sexes. Sex does not have a direct effect on the disease we studied, so it was not considered in the design and analysis.
Field-collected samples	This study does not involve field-collected samples.
Ethics oversight	All animal experiments were conducted with the permission of the Institutional Animal Care and Use Committee of the Qingdao Medical Collage of Qingdao University, China (Approval NO. QDU-AEC-2023269). The present study with patients' informed consent was approved by the Ethics Committee of the Affiliated Hospital of Qingdao University, China.

Note that full information on the approval of the study protocol must also be provided in the manuscript.
