# Supplementary material for: A self-stabilized and water-responsive deliverable coenzyme-based polymer binary elastomer adhesive patch for treating oral ulcer
Source: Nat Commun. 2023 Nov 24;14:7707. doi: 10.1038/s41467-023-43571-x (PMC10673908; doi:10.1038/s41467-023-43571-x)
Supplement: Supplementary file 3 — Description of Additional Supplementary Files [file 41467_2023_43571_MOESM3_ESM.pdf]

Title: Supplementary Movie 1
Description: 3D PCoA of oral flora at the genus level.
